# Neuropeptide changes in an improved migraine model with repeat stimulations

**DOI:** 10.1515/tnsci-2020-0201

**Published:** 2021-12-09

**Authors:** Yichen Guo, Yawen Cheng, Jiaqi An, Yi Qi, Guogang Luo

**Affiliations:** Stroke Center, Department of Neurology, The First Affiliated Hospital of Xi’an Jiaotong University, No. 277 Yanta West Road, Xi’an, 710061, China

**Keywords:** migraine, neuropeptides, electrical stimulation, trigeminal ganglia

## Abstract

Migraine is a medical condition with a severe recursive headache. The activation of the trigeminovascular system is an important mechanism. The neuropeptide calcitonin gene-related peptide (CGRP) plays a crucial role in the pathogenesis of migraine. Several other neuropeptides are also involved; however, their roles in migraine remain unclear. In this study, using a rat model of migraine induced by electrical stimulation of the trigeminal ganglia (TG) and an improved version induced with repeated stimulation, we observed the dynamic changes of these peptides in TG and blood. We demonstrated that the expression of CGRP, pituitary adenylate cyclase activating polypeptide (PACAP), neuropeptide Y (NPY), vasoactive intestinal peptide, and nociceptin in TG was significantly elevated and peaked at different time points after a single stimulation. Their levels in the blood plasma were significantly increased at 12 h after stimulation. The peptides were further elevated with repeated stimulation. The improved rat model of migraine with repeated stimulation of TG resulted in a more pronounced elevation of CGRP, PACAP, and NPY. Thus, the dynamic changes in neuropeptides after stimulation suggest that these neuropeptides may play an important role in the pathogenesis of migraine. Additionally, the migraine model with repetitive stimulation would be a novel model for future research.

## 1 Introduction

Migraine is a severe neurological disorder. The prevalence is about 14.4% [1], affecting about 16% of the adult population. The World Health Organization ranks it as the seventh most disabling disease [2]. However, the pathogenesis of migraine is still unclear. The main theories for the etiology of migraine are (i) vascular, (ii) neuronal, and (iii) neurovascular. The theory of vascular origin, which was first proposed in 1960 [3], posits that during the painful period of vascular headache, a type of migraine, the large and small blood vessels inside and outside the skull are dilated and heavier on one side. The theory of cortical spreading depression (CSD) suggests that migraine attacks involve neurological changes as well as changes in blood flow rather than direct contraction of blood vessels and that migraine aura symptoms are caused by CSD [4]. In this study, we mainly focused on the trigeminal vascular system (TVS) activation theory [5], which also suggests that neuropeptides play an important role in the pathogenesis of migraine. It has been proposed that intracranial blood vessels, especially those of the dura mater, are widely innervated by unmyelinated C-type fibers from trigeminal nerve branches. When they are subjected to electrical, chemical, or other types of noxious stimulation, the nerve terminals release vasoactive neuropeptides. These neuropeptides can cause vasodilation and increase vascular permeability around the dura, leading to sterile inflammation reactions, such as increased plasma protein exudation and mast cell degranulation [6,7]. Inflammation produces antidromic and orthodromic stimuli through the trigeminal nerve. The retrograde stimuli increase the expression and release of neuropeptides, causing or exacerbating headaches. The anterograde stimuli increase the production of c-Fos protein in the trigeminal nucleus and activate the autonomic nervous system, along with other factors, such as calcitonin gene-related peptide (CGRP), causing nausea and vomiting. The neuropeptides described in this process mainly refer to CGRP, which has a powerful vasodilation effect [8].

CGRP is composed of 37 amino acids [9] and is mainly expressed in the nervous system [10]. Studies have shown that nerve fibers around the blood vessels in the brain and nearly half of the neurons in the human trigeminal ganglia (TG) contain CGRP [11]. In the 1990s, it was proposed that CGRP plays an important role in the pathological mechanism of migraine [12,13,14]. CGRP antibodies and CGRP receptor antagonists for migraine treatment have also achieved good therapeutic results [15,16]. CGRP is currently the only neuropeptide confirmed to play a crucial role in the pathogenesis of migraine. It also has a close relationship with CSD, a novel mechanism for migraine origination, which eventually leads to TVS activation [17]. Several other neuropeptides involved in migraine, such as pituitary adenylate cyclase activating polypeptide (PACAP), vasoactive intestinal peptide (VIP), neuropeptide Y (NYP), and nociception, have also been studied. Among them, PACAP and its selective PAC1 receptor also play important roles in the pathogenesis of migraine. In fact, PACAP-PAC1 receptor signaling has become a promising target in migraine therapy [18]. However, the roles of these neuropeptides in migraine are not firmly established and remain to be explored [19,20].

There are many types of migraine animal models [21]. Electrical stimulation of the TG model [22,23] can directly activate the trigeminal nerve, leading to neurogenic inflammation in the distribution area of the nerve to activate TVS, producing migraine-like attacks. Using this animal model of migraine, we observed dynamic changes of the expression of CGRP, PACAP, NYP, VIP, and nociceptin in the TG and the levels of these neuropeptides in jugular vein blood at different time points after stimulation to set a foundation for future studies on the role of these neuropeptides in the pathogenesis of migraine. Furthermore, we improved the current migraine model by repeated stimulation of TG for three consecutive days, which resulted in a more pronounced change in CGRP. Using this improved model, we further evaluated the changes in other promising neuropeptides during a migraine attack.

## 2 Materials and methods

### 2.1 Animals

Male Sprague–Dawley rats, 300–400 g, were purchased from the Animal Experimental Center of Xi’an Jiaotong University Health Science Center. All rats were housed with free access to normal food (Qinle, Xi’an, China) and water, with the temperature controlled at 22 ± 1°C.


**Ethical approval:** The research related to animals’ use has been complied with all the relevant national regulations and institutional policies for the care and use of animals and has been approved by the Committee on Animal Research at Xi’an Jiaotong University and complied with the Guidelines for the Care and Use of Animals [XJTULAC20131068].

## 3 Electrical stimulation of TG migraine model

According to the time of sacrifice after stimulation, the experimental animals were randomly divided into nine groups (*n*  =  6 in each group): the control (C) group, the 0 h (sacrificed immediately after stimulation, S0) group, the 12 h poststimulation (sacrificed at 12 h after stimulation, S12) group, the 24 h poststimulation (sacrificed at 24 h after stimulation, S24) group, sham-stimulation control groups for each of the stimulation groups (N0, N12, N24), the multiple electrical stimulation (MS0) group, and the multiple sham-stimulation (MN0) group. Since the model induces pain in animals, the number of rats used was the minimum necessary to achieve sufficient statistical power. Experimental animals were assigned to receive different treatments, with six rats in each group.

Each rat was anesthetized with sodium pentobarbital (50 mg/kg, i.p.), and the head was fixed in a stereotactic device. After the midline incision, the soft tissue was scraped to expose the surface of the skull, and the anterior condyle was marked. The stimulation electrode was positioned with a stereotactic device (NARISHIGE, SN-2N stereo orientation instrument) 3.2 mm posterior and 3.0 mm lateral to the bregma in the skull on the stimulating site (right side), and a hole with a depth of 9.4 mm was drilled in the skull. The concentric circular electrode (MICROPROBES, WE-3CEA5SS) was slowly inserted to the depth. The stimuli were provided through a concentric circular electrode-wire-current output device (NIHON KONDEN) with an output current of 1 mA, 200 ms cycle, and 5 ms wave width, applied continuously for 30 min [23]. For the sham-stimulation group, only the electrode was inserted into the drilled hole for 30 min without stimulation. After stimulation, the electrode was slowly removed, and the scalp incision was sutured. The wound was covered with erythromycin ointment. The rats remained in a lateral position until they woke from anesthesia. For the multiple electrical stimulation (MS0) group, the TG of the rats was repeatedly stimulated three times using the same parameters, with an interval of 24 h between stimulations. The multiple stimulation control group (MN0) only received three insertions of the electrode without stimulation.

### 3.1 Behavioral observations in rats after electrical stimulation of TG

The rats were placed in a large cage alone, so that they had sufficient freedom for long range movement. There was no noise and odor interference around them, and the temperature and humidity were controlled. There were three observation periods: before and at 24 h after stimulation. The observations included the number of head scratches, cage climbing, excessive hair grooming, and 24 h food intake. The behavioral changes were compared between S24 and N24 groups.

### 3.2 Tissue collection and blood sampling

Rats in different groups were euthanized at 0, 12, and 24 h after stimulation. First, the blood was sampled from the jugular vein using a sterile procedure. Blood samples were collected in tubes with or without heparin. After centrifugation at 2,000 *g* for 15 min, plasma or serum was isolated into 200 µL polypropylene tubes and stored at −80°C for enzyme-linked immunosorbent assays (ELISAs). All samples were confirmed to be free of hemolysis by visual inspection.

After blood extraction, the rats were perfused transcardially with 4°C normal saline with pH 7.0. The TG on the stimulation side was isolated on ice after decapitation then flash frozen in liquid nitrogen and stored at −80°C until further use.

### 3.3 Reverse transcriptase polymerase chain reaction (RT-PCR)

The total RNA of the TG was extracted using Trizol reagent according to the manufacturer’s instructions. The first-strand cDNA was synthesized using a Reverse Transcription-PCR kit (Roche Applied Science, Germany). RT-PCR was performed using a SYBR Green kit (Takara, Japan) in an iCycler iQ5 real-time PCR detection system (BIO-RAD, California, USA). The thermocycling conditions were 50°C for 2 min and 95°C for 10 min, followed by 40 cycles of 95°C for 15 s and 60°C for 1 min. Glyceraldehyde 3-phosphate dehydrogenase (GAPDH) was used as an internal control. The specific primers are as follows: CGRP_forward 5′-CCTGGTTGTCAGCATCTTGCTC-3′, CGRP_reverse 5′-TGCACCAGTGCAGCCAGTA-3′, PACAP_forward 5′-TCCAGCGCAGAAACTCGAAG-3′ PACAP_reverse 5′-TGCATTATTATCCCGTAGACCAACA-3′, NPY_forward 5′-TCCGCTCTGCGACACTACATC-3′, NPY_reverse 5′-AAGGGTCTTCAAGCCTTGTTCTG-3′, VIP_forward 5′-TCAGTTCCTGGCGATCCTGAC-3′, VIP_reverse 5′-CTCCGCTAAGGCATTCTGCAA-3′, nociception_forward 5′-TCTGCACCAGAATGGTAATGTGTAG-3′, nociception_reverse 5′-GGTCTTGGTGTGGACACATGCT-3′, GAPDH_forward 5′-GTCCACGATGAGGACAATGAG-3′, and GAPDH_reverse 5′-CGGCATGTCAGATCCACAAC-3′.

### 3.4 Western blot (WB)

For western blotting, TG tissues were homogenized in radioimmunoprecipitation assay buffer (P0013B, Beyotime, Shanghai, China) with a complete protease inhibitor cocktail (Roche, Germany). After centrifugation, the supernatant was collected, and the protein concentration was measured with bicinchoninic acid (BCA) protein assay kits (P0010, Beyotime, Shanghai, China). A total of 40 µg of protein for each sample was separated by sodium dodecyl sulfate polyacrylamide gel electrophoresis (SDS-PAGE) and electrotransferred onto polyvinylidene fluoride membranes (Millipore, USA).

Membranes were then blocked with 5% nonfat milk for 2 h and incubated with primary antibodies overnight at 4°C. The following primary antibodies were used: CGRP (1:500, Beyotime, AF6495), PACAP (1:1,000, abcam, ab181205), NPY (1:1,000, CST, #11976), VIP (1:500, Beyotime, AF8331), and nociception (1:500, abcam, ab216413). The next day, the membranes were washed, and then incubated with the corresponding secondary antibody (1:10,000; Abmart) for 1 h. The immunoblot bands were detected using an enhanced chemiluminescence system according to the manufacturer’s instructions. GAPDH antibody (1:3,000; Zhuangzhi Bio, China) was used as the internal control. The gray scale of the bands was quantified using Image Lab software version 4.0 (Bio-Rad Laboratories, USA).

#### 3.4.1 ELISA

The levels of plasma neuropeptides were measured by ELISA using the protocol from the manufacturer (ELISA, Yuanye, Shanghai, China) and quantified on a Rayto RT-6000 analyzer (Rayto, Shenzhen, China) at 450 nm. Each sample or standard was measured in triplicate.

### 3.5 Statistical analysis

Statistical analyses were performed using the SPSS 13.0 software. Data in this study were presented as the mean ± SEM. The unpaired Student’s *t*-test was used for comparisons between two groups. The statistical significance level was set at *P* < 0.05 for two-sided tests. Graphs were plotted with GraphPad Prism version 5.01 for Windows (GraphPad Software, USA).

## 4 Results

### 4.1 Electrical stimulation of TG rats evokes migraine-like behavior

**Figure 1:**
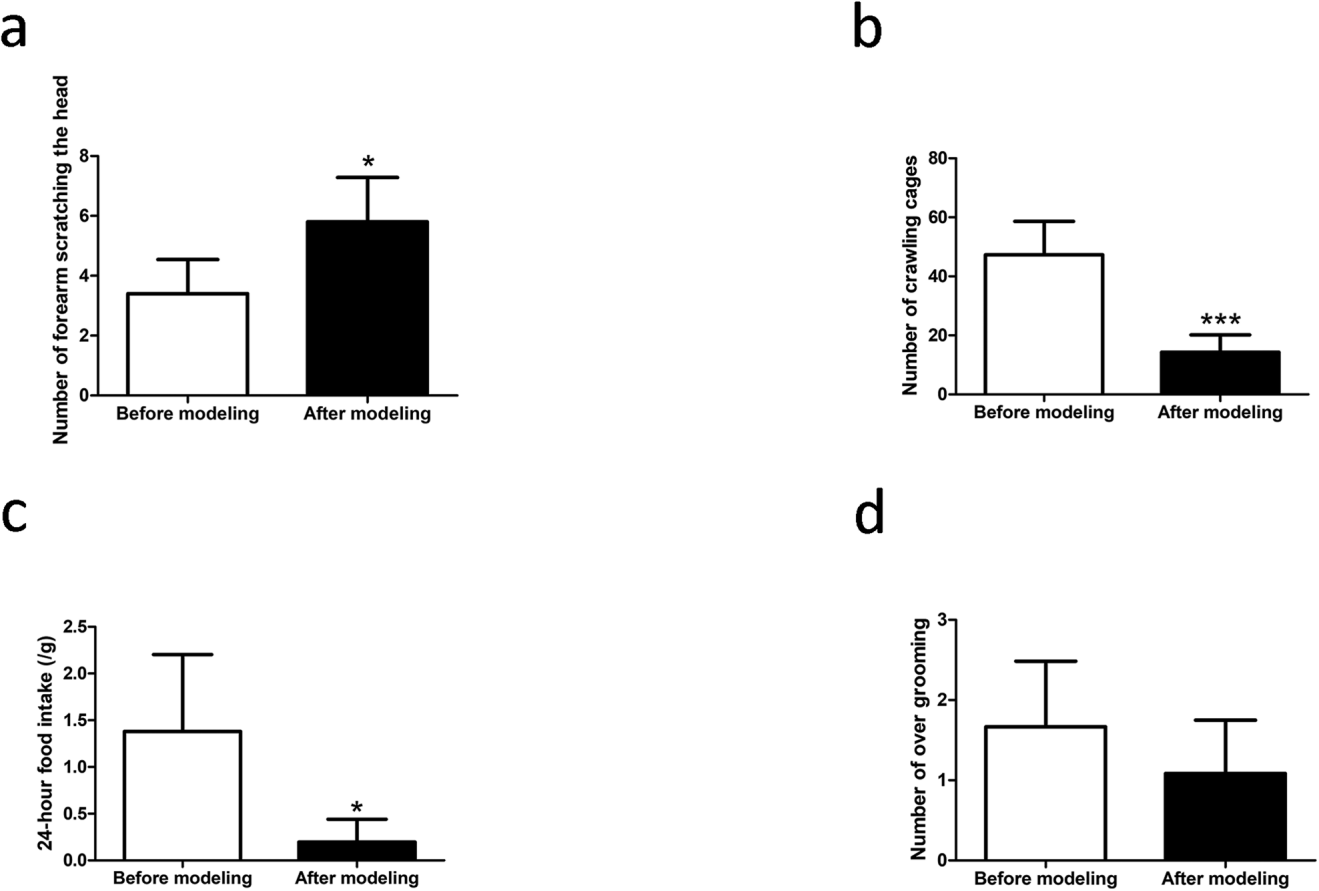
Electrical stimulation of TG rats evokes migraine-like behavior. (a) The number of head scratches. (b) The number of cage crawling events. (c) 24 h food intake. (d) The number of over grooming incidents. Data are shown as the means ± SEM. *n* = 6, *P < 0.05, and ***P < 0.001 vs before modeling.

After the electrical stimulation of the TG, the number of head scratches increased significantly (before: 3.50 ± 0.50 and after: 5.80 ± 0.66, *P* = 0.0209), whereas the number of cage-crawling incidents (before: 47.33 ± 11.29 and after: 14.33 ± 5.85, *P* < 0.0001) and 24 h food intake (before: 1.38 ± 0.82 and after: 0.20 ± 0.24, *P* = 0.0330) decreased significantly. However, there was no statistical difference in the number of over grooming incidents (before: 1.67 ± 0.81 and after: 1.08 ± 0.66, *P* = 0.2045; Figure 1). These changes suggest that the migraine model was successful.

### 4.2 Electrical stimulation of TG upregulates the expression of the neuropeptides and increases their release into the blood

**Figure 2:**
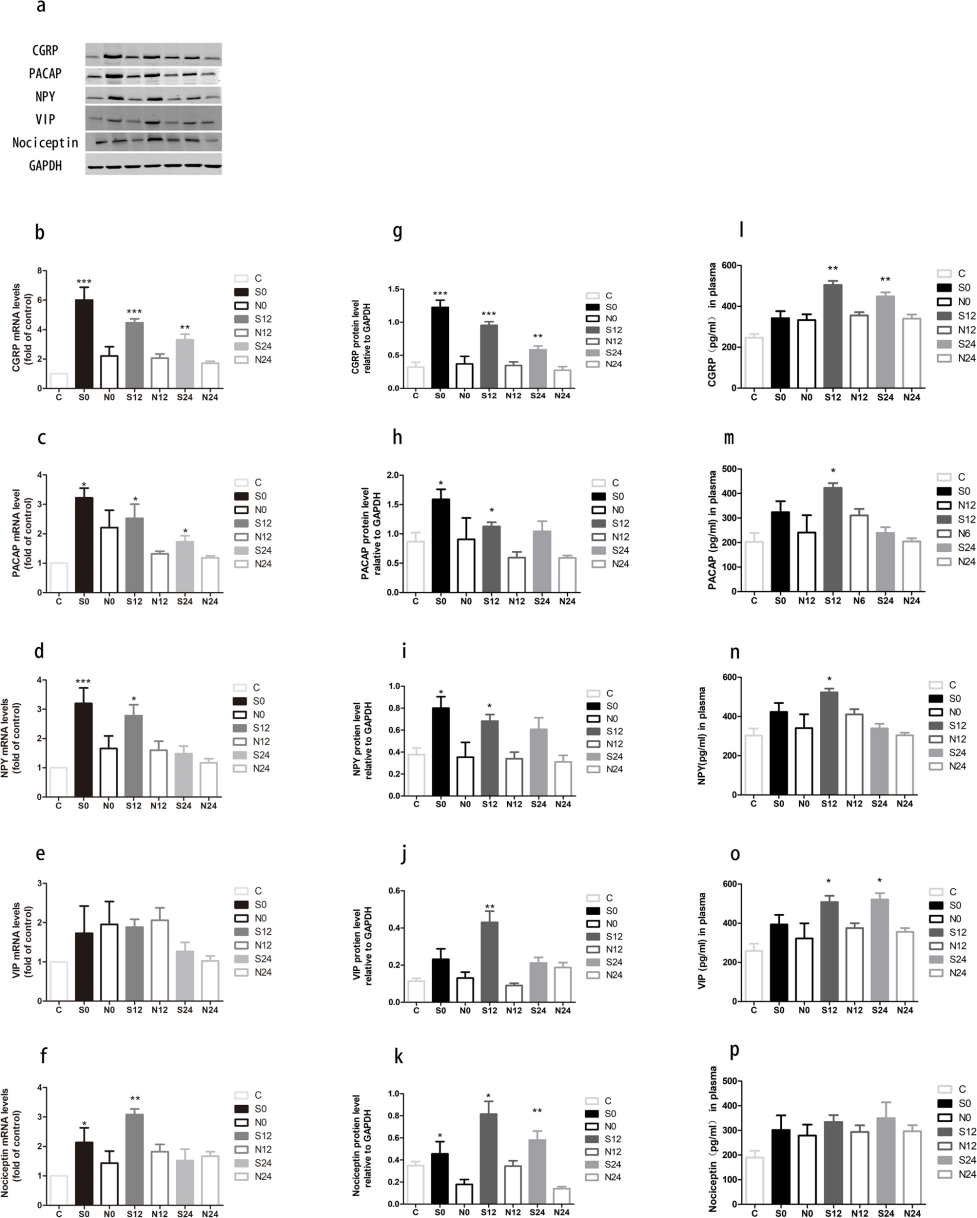
Electrical stimulation of TG upregulates the expression of neuropeptides and increases their release into the blood. (a–e) mRNA levels in the stimulation and sham-stimulation groups measured with RT-PCR. The protein expression in the rat TG (f) and the quantified levels (g–k) detected with WB. (l–p) Neuropeptide levels in plasma were detected with ELISA (C, control; S, stimulation; N, sham-stimulation). Mean ± SEM, *n* = 6 (*vs the sham-stimulation group, *P < 0.05, **P < 0.01, and ***P < 0.001).

To investigate the dynamic changes of the neuropeptides in the rat model of migraine, we measured the mRNA and protein expression of CGRP, PACAP, NPY, VIP, and nociceptin in the TG on the stimulated side and the protein levels of these neuropeptides in the plasma. As shown in Figure 2a, the mRNA level of CGRP increased in the stimulated group compared to that in the sham control (*P*_S0_ < 0.0001, *P*_S12_ = 0.0002, *P*_S24_ = 0.0048) and reached the highest value immediately after stimulation. At the protein level (Figure 2f), the CGRP concentration in the stimulated group was significantly higher than that in the sham-simulation group, as revealed by WB analysis (Figure 2g; *P*_S0_ < 0.0001, *P*_S12_ = 0.0003, *P*_S24_ = 0.0077) and ELISA (Figure 2l; *P*_S0_ = 0.0012, *P*_S12_ = 0.0084).

With respect to PACAP, the mRNA expression increased after stimulation when compared to the expression in the sham-stimulation group (Figure 2b; *P*_S0_ = 0.0103, *P*_S12_ = 0.0389, *P*_S24_ = 0.0365) and reached the highest value immediately after stimulation. Similar results were observed for its protein expression. WB analysis revealed that the PACAP protein level increased in rat TG after stimulation (Figure 2f and h; *P*_S0_ = 0.0433, *P*_S12_ = 0.0119). As Figure 2m shows, the ELISA results showed a similar change (*P*_S12_ = 0.0263).

Regarding the NPY, the mRNA expression increased after stimulation compared to the expression in the sham-stimulation group (Figure 2c; *P*_S0_ = 0.0010, *P*_S12_ = 0.0408) and reached the highest value immediately after stimulation. WB results revealed that the protein level of NPY was significantly elevated (Figure 2f and i, *P*_S0_ = 0.0106, *P*_S12_ = 0.0158) after stimulation. This trend was in accordance with the changes observed in the ELISA results (Figure 2n, *P*_S12_ = 0.0263).

As shown in Figure 2d, VIP mRNA expression was not significantly different between the stimulation and sham-stimulation groups at any time point. However, the VIP protein level in the 12 h group after stimulation was higher than that in the sham-stimulation group (Figure 2f, j. *P*_S12_ = 0.0158) by WB. ELISA analysis showed a similar change (Figure 2o; *P*_S12_ = 0.0305, *P*_S24_ = 0.0121).

With respect to nociceptin, the mRNA expression increased after stimulation compared with the expression in the sham-stimulation group (Figure 2e; *P*_S0_ = 0.0399, *P*_S12_ = 0.0037) and reached the highest value at 12 h after stimulation. This trend was in accordance with the WB results. WB analysis revealed that the nociceptin protein levels increased in rat TG after stimulation (Figure 2f and k; *P*_S0_ = 0.1159, *P*_S12_ = 0.0194, *P*_S24_ = 0.0063). However, the ELISA results showed no significant difference between the stimulation and sham-stimulation groups.

### 4.3 Repetitive stimulation of rat TG further enhances neuropeptide expression

**Figure 3:**
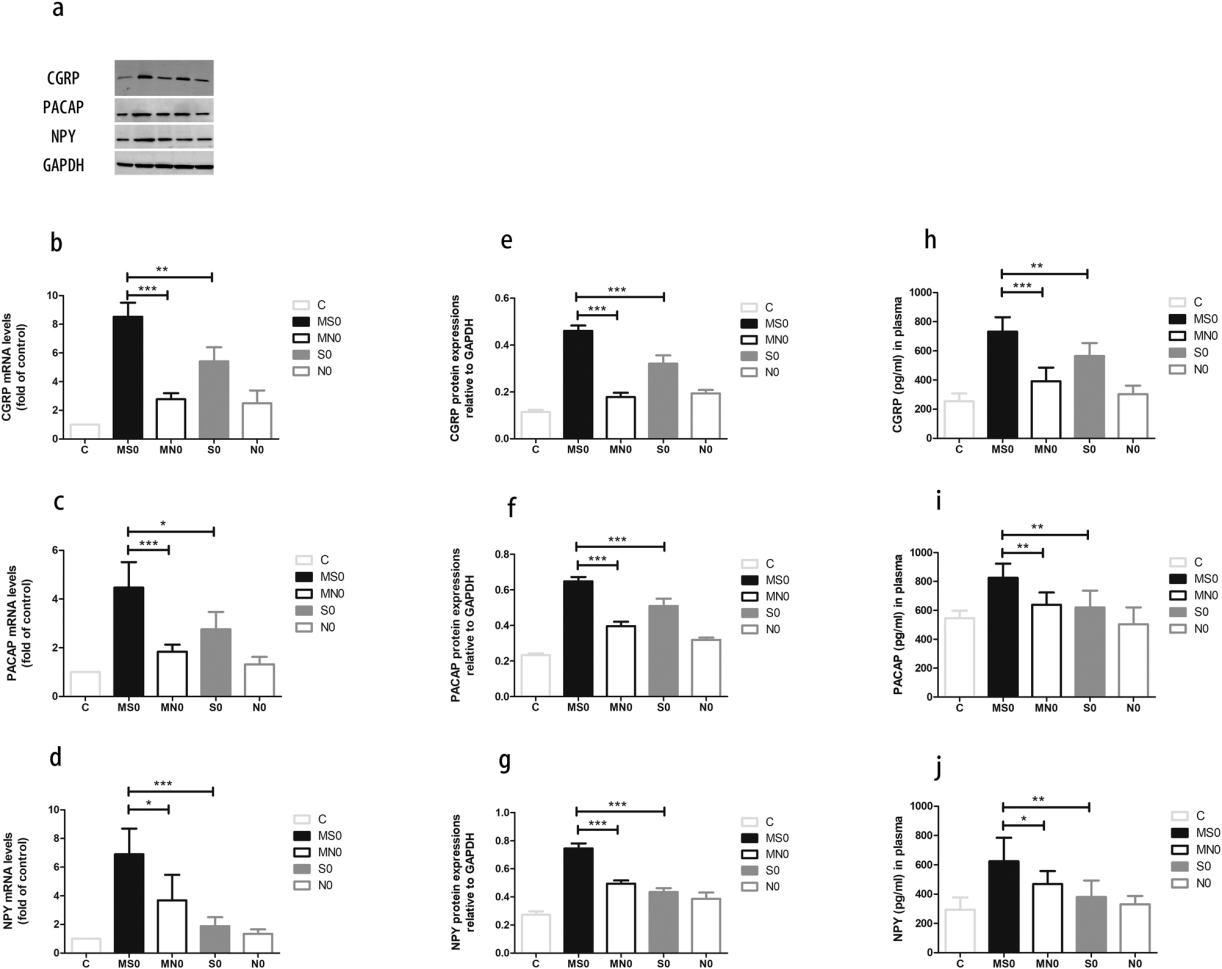
Repetitive stimulation of rat TG further enhances neuropeptide expression. (a–c) mRNA levels measured with RT-PCR. (d–g) Protein expression in the rat TG (d) and the quantified levels (e–g) detected with WB. (h–j) Neuropeptides levels in plasma levels were detected with ELISA (C, control; S, stimulation; N, sham-stimulation; MS, multiple stimulation; MNS, nonmultiple stimulation). Mean ± SEM, *n* = 6. (*P < 0.05, **P < 0.01, and ***P < 0.001).

To establish an animal model to mimic periodic migraine attacks, we repeated the electrical stimulation of TG three times over a 3 day period, with an inter-stimulation interval of 24 h. As shown in Figure 3a, the CGRP mRNA level in the repeated stimulation group was significantly higher than that in the single stimulation group (*P* = 0.0011). A similar increase in the protein expression level of CGRP was also observed in the repeated stimulation group, as shown by WB (Figure 3d and e; *P* < 0.0001) and ELISA (Figure 3h) results (*P* = 0.0061). Further enhancement of the expression of CGRP in TG by multiple stimulation suggests that this novel method of stimulation can produce a better animal model for migraine. Thus, we used this model to further evaluate the changes in other peptides after repeated stimulation. As shown in Figure 3b–j, the expression levels of both PACAP and NPY were further increased above the level by single stimulation (PACAP: *P* = 0.0167, *P* = 0.0002, and *P* = 0.0038; NPY: *P* = 0.0004, *P* < 0.0001, and *P* = 0.0065). Thus, we have more confidence to conclude that the PACAP and NPY expression levels are significantly enhanced in the migraine model.

## 5 Discussion

Migraine is a comprehensive process involving both vascular and neurological factors. Electrical stimulation of the rat TG can directly activate TVS and produces a reasonably good migraine model [23,24]. Many studies have shown that migraine models [25,26,27] can cause behavior changes in rats. The neuropeptide CGRP plays a crucial role in the pathogenesis of migraine. Several other neuropeptides are also involved in migraine. However, changes in these neuropeptides during migraine attacks and their roles in migraine development remain to be explored. In this study, using a migraine model developed with electrical stimulation of TG and an improved version of the model, we demonstrated that the expression levels of CGRP, PACAP, NPY, VIP, and nociceptin in TG were significantly increased immediately after single stimulation and gradually recovered over 24 h. However, repeated stimulation for 3 consecutive days further exacerbated the changes in these neuropeptides. Thus, we confirmed that there is a significant increase in these peptides in an improved animal model of migraine.

For behavior measurement, since the tissues were extracted immediately after modeling for the S0 group and the rats had not fully recovered from anesthesia for the S6 group, we chose only the S24 group to observe the number of forelimb scratches, cage climbing, excessive grooming, and 24 h food intake. After stimulation, the number of head scratches was significantly higher, the number of cage-climbing events and 24 h of food intake were significantly reduced, and the number of excessive grooming incidents was not significantly different. In this process, persistent damage of the TG caused physiological discomfort in rats. Some rats changed from a manic hyperactivity state to a depression state, resulting in a decrease in the number of cage-climbing incidents and food intake. In addition, incomplete recovery from the long-lasting effects of the anesthesia on muscles can also result in a significant reduction in the activities of rats. Interpretation of the above behavioral changes requires further expansion of the sample size, a normative observational environment, and adoption of more objective behavioral observation indicators.

The increase in the expression of the vasoactive neuropeptides in TG and their release (as indicated by the increase of their level in the blood) suggest that they are potentially involved in the pathogenesis of migraine. According to the TVS activation theory, the neurons in the TG are activated by various factors, and vasoactive peptides are retrogradely released along the V1 branch of the trigeminal nerve. A neurogenic inflammatory response then occurs, leading to strong intracranial vasodilation and increased plasma protein extravasation. Astrocytes in TG are also activated at the same time, and positive feedback releases inflammatory factors such as interleukin (IL)-1, IL-6, tumor necrosis factor-α, histamine, serotonin (5-HT), and other chemokines, further aggravating the TVS-derived inflammatory response. The cascade of stimuli is transmitted to the cortical sensory center through multilevel neurons, activating the pain modulation nucleus, such as the blue nucleus in the brain, sensitizing the peripheral receptors, and resulting in a strong pulsating headache [28,29]. In this process, neuropeptides, such as CGRP, PACAP, NPY, VIP, and nociception, may be involved in neurogenic inflammation of the intracranial vasculature (plasma protein extravasation and vasodilation) and peripheral and central sensitization of the trigeminal nervous system [30].

CGRP has been widely regarded as an important neuropeptide in the pathophysiology of migraine [31]. The mechanism of PACAP’s role in migraine has also gradually attracted more attention. Studies have shown that the injection of PACAP-38 can produce obvious migraine-like seizures and continuous dilatation of extracranial arteries in patients with migraine [18] and may directly sensitize the trigeminal sensory fibers [32]. It should be noted that the form of PACAP detected in our WB was the precursor, preproPACAP. An increase in the precursor protein would also result in elevation of the major active form PACAP (PACAP-38), although we cannot exclude the possibility that the minor form PACAP-27 was also different in the migraine model. However, the changes in NPY [14,33,34,35,36], VIP [14,37,38,39,40], and nociception [41,42,43] during migraine attacks and their relationship with migraine are still controversial.

In this study, CGRP, PACAP, NPY, VIP, and nociceptin were recorded at different times after stimulation to explore the dynamic changes in the neuropeptides during migraine attacks. The results showed that except for VIP, the mRNA expression levels for all of the other four neuropeptides increased after electrical stimulation. Similar changes in these peptides were also observed in the protein levels. Interestingly, CGRP, PACAP, and NPY in the TG increased rapidly after stimulation, gradually decreased with time, and returned to normal within 24 h after stimulation. In contrast, VIP and nociceptin increased more slowly after stimulation and peaked in 12 h. In jugular vein blood, the rate of the change of these peptides at the protein level was slower than that of the TG and gradually reached its peak after 12 h of stimulation. This result suggests that it takes some time for neuropeptides to be released into peripheral blood. The changes in neuropeptides at different rates in the TG and peripheral blood suggest that the electrical stimulation of the TG model leads to an increase in the level of major neuropeptides in TVS and gradually decreases after they are released into the blood. Collectively, we propose that PACAP, NPY, VIP, and nociceptin may play important roles in the pathogenesis of migraine. They are also potential new targets for migraine treatment.

TG stimulation is a well-established method for activating the TVS [44]. When unilateral TG electrical stimulation is performed, the oral and nasal secretions on the stimulated side are increased, and the masticatory and eye muscles contract. These phenomena are indicators that the electrodes have effectively stimulated the TG, especially the branch of the eye, which is the main branch for pain transmission. It also suggests that the electrical stimulation of TG results in TVS activation.

Periodic attacks are a typical feature of migraine [44]. A single stimulation can only simulate a single acute attack of migraine. To produce a migraine model with periodic attacks, we repeatedly applied electrical stimulation of TG for three consecutive days with the same stimulation parameters as the traditional electrical stimulation methods [23]. As mentioned above, the CGRP is a well-established neuropeptide associated with acute migraine attacks [12,13,14] and its concentration correlates with the timing and severity of a migraine [45]. Meanwhile, our results showed that the expression levels of PACAP and NPY also peaked immediately with single stimulation. We evaluated this model based on the expression of CGRP, PACAP, and NPY. Our results that the expression of these neuropeptides in the repetitive stimulation group (MS0) significantly increased, when compared to both the sham-stimulation group (MN0) and single stimulation group (S0). Thus, repeated stimulation further validated our results for single stimulation with more confidence. During the stimulation process, there were contractions of the chewing muscles on the stimulating side and the increase of oral and nasal secretions. Therefore, the results suggest our repetitive stimulation TG model exhibits some characteristics of migraine. More importantly, repetitive stimulation is similar to the repeated occurrence of migraine. Taken together, our novel repetitive stimulation TG model appears to be a better migraine model.

## 6 Conclusion

The dynamic changes in neuropeptides after stimulation suggest that CGRP, PACAP, NPY, VIP, and nociceptin may play a role in the pathogenesis of migraine. We have developed a novel rat model of migraine with repeated electrical stimulation of the TG. Repetitive stimulation can aggravate the expression of neuropeptides, suggesting it is a better migraine model.
